# SNX-2112 Induces Apoptosis and Inhibits Proliferation, Invasion, and Migration of Non-Small Cell Lung Cancer by Downregulating Epithelial-Mesenchymal Transition via the Wnt/β-Catenin Signaling Pathway

**DOI:** 10.7150/jca.56640

**Published:** 2021-08-03

**Authors:** Xiaozhen Cheng, Lingyu Qin, Lian Deng, Xiongjie Zhu, Ying Li, Xiaoran Wu, Yanfang Zheng

**Affiliations:** 1 Medical Oncology Department, Affiliated Cancer Hospital & Institute of Guangzhou Medical University, No.78 Heng-Zhi-Gang Road, Yue Xiu District, Guangzhou 510095, China.; 2 Oncology Center, Zhujiang Hospital, Southern Medical University, No. 253 Industry Road, Guangzhou 510282, China.; 3 Department of Oncology, Central South University Xiangya School of Medicine Affiliated Haikou Hospital, Hainan Province, 570208, China.

**Keywords:** non-small cell lung cancer, SNX-2112, epithelial-mesenchymal transition, Wnt/β-catenin

## Abstract

Lung cancer is the most frequent malignant tumor, and non-small cell lung cancer (NSCLC) is responsible for substantial mortality worldwide. The small molecule SNX-2112 was recently shown to critically effect the proliferation and apoptosis of tumor cells. Nevertheless, the precise mechanism by which SNX-2112 affects NSCLC remains poorly understood. Therefore, we investigated the function of SNX-2112 in NSCLC. We verified that SNX-2112 promoted apoptosis and suppressed the proliferation, invasion, and migration of A549 and H520 NSCLC cells *in vitro*. We further verified the potential mechanism of SNX-2112 in NSCLC. The changes in the protein levels demonstrated that SNX-2112 inhibited the epithelial-mesenchymal transition (EMT) (increased E-cadherin and decreased N-cadherin and vimentin) and the Wnt/β-catenin signaling pathway (glycogen synthase kinase (GSK) 3β and phosphorylated (p)-β-catenin increased, β-catenin and p-GSK3β decreased) in NSCLC cells. These results were verified by rescue experiments using a Wnt/β-catenin pathway agonist. We also established a tumor xenograft model and confirmed that SNX-2112 reduced tumor growth and proliferation and enhanced necrosis and apoptosis in a NSCLC model *in vivo*. In conclusion, the current study is the first to discover the mechanism of SNX-2112 in NSCLC. SNX-2112 induced apoptosis and also inhibited the proliferation, invasion, and migration of NSCLC cells by downregulating EMT via the Wnt/β-catenin signaling pathway.

## Introduction

Lung cancer is the most common malignant tumor with the highest morbidity and mortality rates worldwide 1. Despite substantial progress in cancer therapies, the five-year survival rate of lung cancer patients remains <20% 2. Molecularly targeted drugs have the advantage of few side-effects and specific patient efficacy, and thus offers new hope to patients with lung cancer. However, patients frequently develop resistance to these drugs after 8-12 months of treatment 3, and tumor recurrence and metastasis remain the main causes of death in lung cancer patients. Therefore, the development of novel drugs targeting tumor recurrence and metastasis continues to be the focus of current cancer treatment.

Heat shock protein 90 (Hsp90) is a molecular chaperone involved in the maintenance of structural and catalytic functions of various proteins in homeostasis. In addition, Hsp90 is involved in key steps in carcinogenesis, including the stabilization of oncogenic proteins, induction of tumor angiogenesis, inhibition of apoptosis, replication, and senescence, as well as promoting tumor invasion and metastasis 4. Previous studies have indicated that Hsp90 was enriched in the tissues and peripheral blood in patients with malignancies, including lung cancer 5, 6, gastric cancer 7, and breast cancer 8. Thus, Hsp90 overexpression could lead to tumor invasiveness and a poor prognosis, while its downregulation has been shown to inhibit tumor growth 9. Multiple studies have shown that Hsp90 can trigger EMT in many solid tumors, including colorectal cancer 10, ovarian cancer, renal cancer 11, and breast cancer 12. In addition, extracellular Hsp90 was found to promote cell motility in an ERK and matrix metalloproteinase-2/9-dependent manner, and shift the cellular morphology toward a mesenchymal phenotype 13. HSP90 also interacts with STAT3 in both the cytoplasm and nucleus to mediate TWIST1 expression. The inhibition of HSP90 downregulates STAT3 activity and TWIST1 transcription, thereby suppressing EMT and potentially inhibiting tumor progression, metastasis, and chemoresistance 11. Hsp90 has also been found to promote sphere formation in cervical cancer 14. Moreover, the Hsp90-induced EMT and tumor stem cell characteristics was associated with the occurrence and development of liver cancer 15.

The small molecule Hsp90 inhibitor, SNX-2112, competitively binds to the ATP site on Hsp90 and induces degradation of the Hsp90 receptor protein, ultimately leading to tumor cell apoptosis 16-18. SNX-2112 has been shown to inhibit the proliferation of multiple tumor cell lines *in vitro*, including malignant melanoma, liver cancer, cervical cancer, and breast cancer cells 19. In addition, SNX-2112 exerted an inhibitory effect on orthotopic tumor xenografts *in vivo*, including liver cancer 20, chronic myelogenous leukemia 21, and melanoma 22. However, the effect of SNX-2112 in NSCLC and the potentially antineoplastic mechanism of SNX-2112 remains unclear and requires further study.

We next sought to reveal the function of SNX-2112 in NSCLC utilizing relevant methods and examine its effects on the levels of EMT and Wnt/β-catenin pathway protein expression, in order to disclose the potential molecular target of SNX-2112. Consequently, this study is the first to suggest a mechanism of SNX-2112 in NSCLC and its effects on EMT and Wnt/β-catenin signaling.

## Materials and methods

### Reagents

Penicillin and streptomycin, RPMI 1640, Dulbecco's Modified Eagle's Medium (DMEM), 0.25% trypsin, 0.25% trypsin-EDTA, and fetal bovine serum (FBS) were obtained from Gibco (Grand Island, NY, USA). Cell Counting Kit-8 (CCK-8) and the Annexin V-FITC/PI Apoptosis Detection Kit were purchased from Dojindo Molecular Technologies, Inc. (Japan). Matrigel was purchased from Becton, Dickinson and Company (Franklin Lakes, NJ, USA). SNX-2112 (PF-04928473; >98%) was purchased from Dalian Meilun Biotechnology Co., Ltd. (Dalian, Liaoning, China). The Wnt/β-catenin pathway agonist, SKL2001, was obtained from Topscience Co., Ltd. (T6989; Shanghai, China). The immunohistochemical staining kit was purchased from Boster Biological Technology Co. Ltd. (SV0002; CA, USA). The following primary antibodies were employed: E-cadherin (rabbit polyclonal, 20874-1-AP; Proteintech, Chicago, IL, USA), N-cadherin (rabbit polyclonal, 22018-1-AP; Proteintech), vimentin (rabbit polyclonal, 10366-1-AP; Proteintech), β-catenin (rabbit monoclonal, mAb #8480S; Cell Signaling Technology, Danvers, MA, USA), phosphorylated (p)-β-catenin (rabbit monoclonal, mAb #8480S; Cell Signaling Technology), glycogen synthase kinase (GSK) 3β (rabbit polyclonal, 22104-1-AP; Proteintech), p-GSK3β (rabbit polyclonal, 14850-1-AP; Proteintech), matrix metalloproteinase (MMP)-9 (rabbit monoclonal, ab76003; Abcam, Cambridge, MA, USA), c-Myc (rabbit monoclonal, ab32072; Abcam), cyclinD1 (rabbit monoclonal, ab134175; Abcam), CD44 (rabbit monoclonal, ab189524; Abcam), Ki-67 (rabbit polyclonal, ab15580; Abcam), glyceraldehyde 3-phosphate dehydrogenase (GAPDH) (rabbit polyclonal, ab9485; Abcam), and survivin (rabbit monoclonal, ab76424; Abcam). Secondary antibodies (goat polyclonal anti-rabbit IgG (H+L) and BA1039) were purchased from Boster Biological Technology Co. Ltd.

### CCK-8 assay

Human NSCLC cell lines, A549 and H520 (ATCC; The Global Bioresource Center, Manassas, VA, USA), were cultivated in DMEM and RPMI 1640 containing 10% FBS, respectively, at 37°C with 5% CO_2_. The tumor cells (approximately 5×10^4^ cells/well) were then transferred into 96-well plates. To explore the IC50 values, we treated the cells with SNX-2112 at 2.15e^-5^, 2.15e^-4^, 2.15e^-3^, 2.15e^-2^, 2.15e^-1^, 2.15, 21.5, and 215 µM and cultured them for 24, 48, and 72 h, respectively. We then added the blended CCK-8 solution to each well at the applicable time and cultured the cells for an additional 1.5 h in a 37°C incubator. The absorbance was detected and measured under 450 nm with a microplate reader (SpectraMax M5, MDS Corporation, CA, USA). The following equation was used to calculate the cell viability (%):


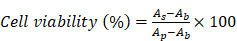


where *A_s_*, *A_b_*, and *A_p_*, refer to the absorbances of the sample, blank control, and positive control, respectively.

### Apoptosis detection

A549 and H520 cells (approximately 2×10^5^ cells/well) were collected and transferred into plates. Furthermore, the cells were cultivated at 37 °C for 24 h. Two milliliters of culture medium containing different concentrations of SNX-2112 or culture medium without SNX-2112 (blank control) were subsequently added to the wells after the tumor cells were fully adhered, followed by incubation for further 48 h. The adherent cells in the remaining medium were carefully resuspended in PBS (pH 7.4, 4 °C) and centrifuged (426.80×*g* for 5 min). The culture medium was discarded, and the cells were subsequently stained in accordance with the manufacturer's instructions in the Annexin V-FITC/PI Apoptosis Detection Kit. The cells were finally examined using a FACScan instrument (Becton, Dickinson and Company).

### Wound-healing assay

Five lines about 1 cm apart were drawn on the bottom of a 6-well plate. A549 and H520 cells were then embedded into the wells and cultured to approximately 100% confluence. The removed cells were washed away using PBS after the plates were scraped using a 20 μL pipette tip. Then, serum-free cell culture medium and SNX-2112 were carefully added to the cells. The cells in each experimental group were observed under a microscope at 0 and 24 h after scratching, and five randomly selected fixed fields of view for each experimental group were photographed and labeled to analyze the wound-healing ability. The healing rate of the cells was indicated by their lateral migration ability. The following equation was applied to calculate the wound-healing rate (%):


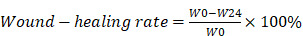


where *W_0_* and *W_24_* refer to the width of the scratches at 0 h and 24 h, respectively.

### Cell migration and invasion assays

SNX-2112's invasive and migratory abilities of the tumor cells were examined using 24-well Transwell chamber assays (Millipore, MA, USA). To assess cell invasion, the inserts were pre-coated with 100 µL Matrigel and A549 and H520 cells (4×10^4^/chamber) treated with different concentrations of SNX-2112 were suspended in serum-free RPMI 1640/DMEM. Next, by adding the tumor cells to the upper chamber, the appended 500 µL RPMI 1640/DMEM supplemented with 10% FBS in the lower chamber was considered as the chemoattractant. Subsequently, the cells were cultivated for 24 (migration) or 36 h (invasion), and the cells that did not migrate or invade were lightly wiped off. The cells that remained under the membrane were fixed in 4% paraformaldehyde for 30 min and stained with 0.1% Crystal Violet (Beijing Legend Biotechnology Co., China) for 15 min. After flushing with running water, we selected five random fields from each group to observe and count the migratory or invaded cells using a microscope.

Rescue experiments with SKL2001 were also performed using a Transwell assay to verify that SNX-2112 inhibited the invasion and migration of NSCLC cells via inhibiting the Wnt/β-catenin pathway. We subsequently divided the cells into four equal groups: 1) a negative control group; 2) cells treated with SNX-2112; 3) cells treated with SKL2001; and 4) cells treated with SNX-2112 and SKL2001. Based on the results of preliminary experiments, A549 cells received 5.38 µM SNX-2112 and 20 µM SKL2001, and H520 cells received 1.35 µM SNX-2112 and 20 µM SKL2001. Their invasion and migration abilities were compared with that of the blank control group. All other procedures were as described above.

### Antibodies and Western blotting

The samples were collected and washed twice with PBS followed by lysis in cold RIPA buffer containing 1% protease inhibitor and a 1% phosphatase inhibitor. After preparing all the necessary procedures, proteins were divided into equal portions (50 μg) using SDS-PAGE and subsequently transferred to the membrane. The level of protein expression was detected using primary antibodies to GAPDH, E-cadherin, N-cadherin, vimentin, β-catenin, p-β-catenin, GSK3β, p-GSK3β, MMP-9, c-Myc, cyclinD1, CD44, and survivin, respectively. Anti-rabbit secondary antibodies were incubated for 1-1.5 h with the membrane. The target proteins were examined using a Tanon Chemiluminescence Meter 5200 (Shanghai Tanon Technology Co., China). The gray value of each strip was analyzed using Image J analysis software. Trends in three replications were similar; thus, we chose the most representative images and exhibited the results in **Figure 6A-C**.

### Animals and *in vivo* tumor xenograft model

All 20 three-week-old male BALB/c nude mice were provided with normal diet *ad libitum* for one week and then subcutaneously injected with 5.5×10^6^ A549 cells/100 µL sterile PBS. When the tumors reached a diameter of 5-10 mm, the mice were distributed randomly among four groups: 1) a saline group; 2) saline group containing 60% dimethyl sulfoxide (DMSO); 3) low-dose SNX-2112 (5 mg/kg) group; and 4) high-dose SNX-2112 (10 mg/kg) group (5 mice per group). The treatments were administered by continuous tail-vein injection for seven days. Due to statistical requirements, the mouse body weight and tumor diameter were gauged every other day. The equation presented below shows that the tumor volume was obtained utilizing the following formula:


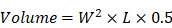


where W and L refer to the average width and length of the solid tumor. All mice were killed humanely by trained certified person using cervical dislocation on day 8, and tumor tissues were collected for further detection *in situ*. The tumor tissues were fixed integrally in 4% paraformaldehyde, embedded in prepared paraffin, and then sliced into 4-μm sections for HE staining, IHC staining, and a TUNEL assay. The Nanfang Hospital Animal Ethics Committee (Guangzhou, Guangdong, China) authorized all experimental protocols involving the use of animals (NFYY-2018-81).

### HE staining

Tissue samples were refrigerated at -20 °C overnight, cut into 4 μm sections, and then fitted onto slides. After incubation at 60 °C for 30 min, the sections were dipped in xylene twice for 30 min, washed twice in 100% ethanol for 10 min, and then washed in 95%, 90%, 80%, and 70% ethanol for 5 min. The slides were subsequently dipped in hematoxylin for 1 min. After flushing in running water, the sections were dipped in eosin for 2 min, dehydrated with ethanol, and sealed with resin.

### IHC

The level of Ki-67 expression in the tumor tissues was determined by IHC. The slides were immersed in xylene for 30 min, hydrated twice in 100% ethanol for 10 min, and then hydrated in 95%, 90%, 80%, and 70% ethanol for 5 min. After washing three times in PBS, antigen unmasking was performed using sodium citrate antigen retrieval buffer (Boster Biological Technology Co. Ltd.). Furthermore, the slides were soaked in boiled buffer, heated in the microwave for 20 min, followed by blocking with 3% H_2_O_2_ and 5% BSA to eliminate the effects of nonspecific antigens. Eventually, the tissues were incubated with a primary antibody against Ki-67 (rabbit anti-Ki67 polyclonal antibody, ab15580; Abcam) at 4 °C overnight. The samples were subsequently cultured with a secondary antibody for another 30 min and finally stained with DAB chromogen (EnVision Detection System kit, Dako, Denmark). The cell nucleus was stained with hematoxylin.

### TUNEL assay

Paraffin blocks containing fixed tumor tissues were sliced into 4 μm pieces and fitted onto slides at 37 °C overnight. The slides were then immersed twice in xylene for 10 min and then washed twice in 100% and 95% ethanol for 5 min. The remaining procedures were performed based on the instructions in the TUNEL kit (Roche, Switzerland). The cell nucleus was dyed with hematoxylin.

### Statistical analysis

Different concentrations were used for individual experiments in order to obtain the optimal concentration. The original data were processed using SPSS 21.0 (SPSS Inc., Chicago, IL, USA) and GraphPad Prism 7.0 Software (GraphPad Software, La Jolla, CA, USA). The results were displayed as the mean ± standard deviation. In general, comparisons of differences between the groups were performed using a one-way ANOVA and the least significant difference method. To reduce the amount of experimental error, all experiments were performed in triplicate. The statistical significance of the results was considered based on a threshold P value <0.05.

## Results

### SNX-2112 inhibited NSCLC cell proliferation

**Figure 1:**
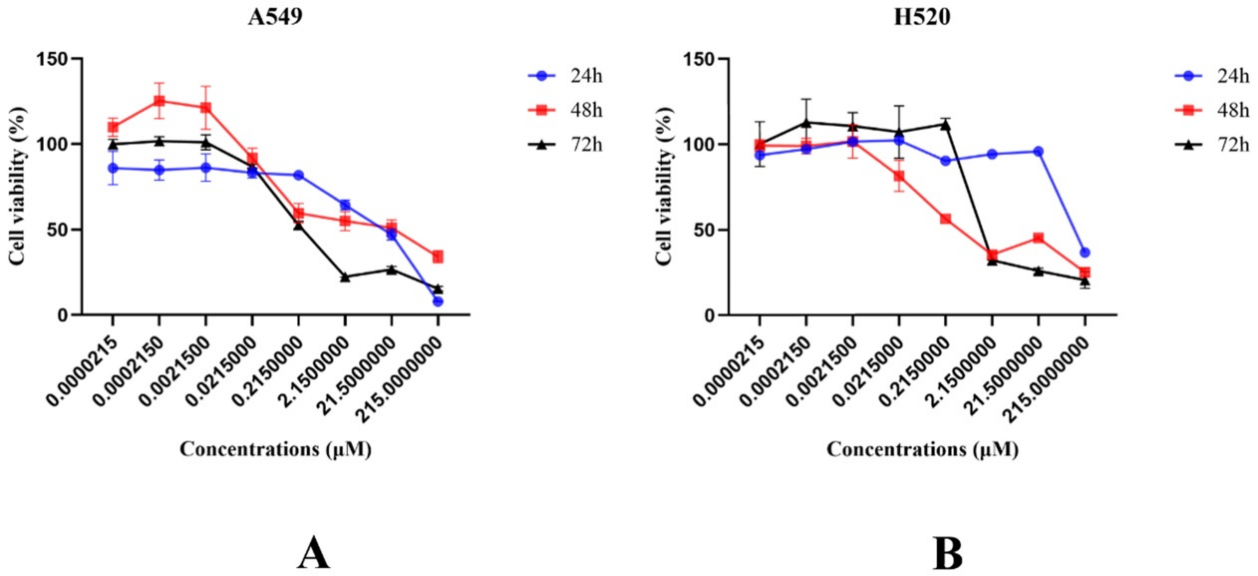
Cell viability declined following treatment with SNX-2112. (A) The viability of A549 cells were examined following the administration of various concentrations of SNX-2112 for 24, 48, and 72 h. (B) The viability of H520 cells were examined following the administration of various concentrations of SNX-2112 for 24, 48, and 72 h.

**Figure 2:**
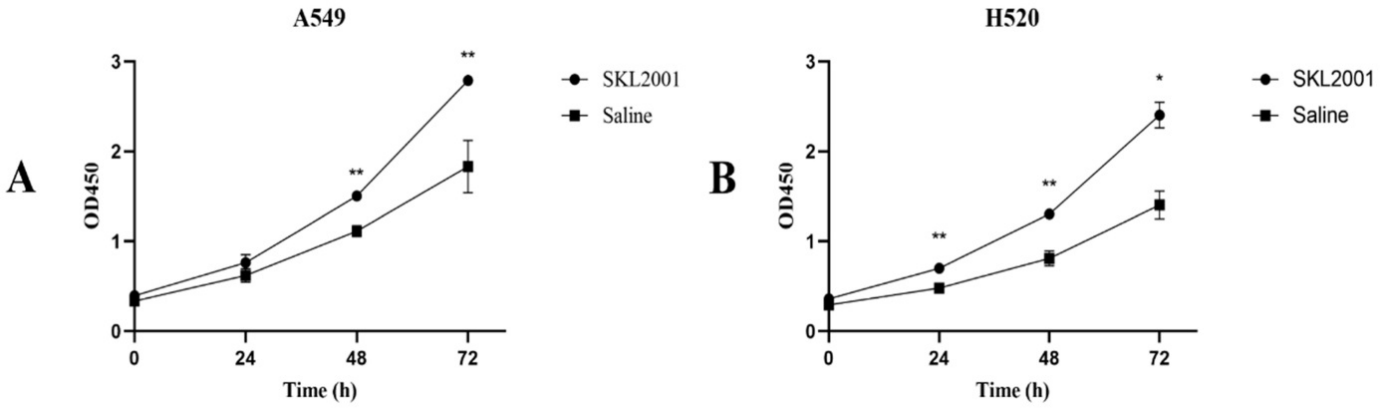
SKL2001 promoted cell proliferation. (A) The viability of A549 cells were measured after administration of 20 µM SKL2001 for 24, 48, and 72 h. (B) The viability of H520 cells were measured after administration of 20 µM SKL2001 for 24, 48, and 72 h (^*^P<0.05; ^**^P<0.01).

Cell viability was evaluated to detect the impact of SNX-2112 on NSCLC cell proliferation. For this purpose, the cell viability was observed to decline significantly with the treatment of SNX-2112 in a dose-dependent manner (**Figure 1A and B**) (P<0.01). We also tested the effects of SKL2001 on cell proliferation utilizing a CCK-8 assay. As shown in **Figure 2A and B**, cellular proliferation was boosted following the administration of 20 µM SKL2001. Taken together, these results provide further confirmation that SNX-2112 might inhibit cellular proliferation by inhibiting the Wnt/β-catenin signaling pathway (P<0.05 and P<0.01). Moreover, the IC_50_ values for SNX-2112 indicated that SNX-2112 suppressed the proliferation of NSCLC cells **(Table 1)**.

### SNX-2112 induced apoptosis *in vitro*

**Figure 3:**
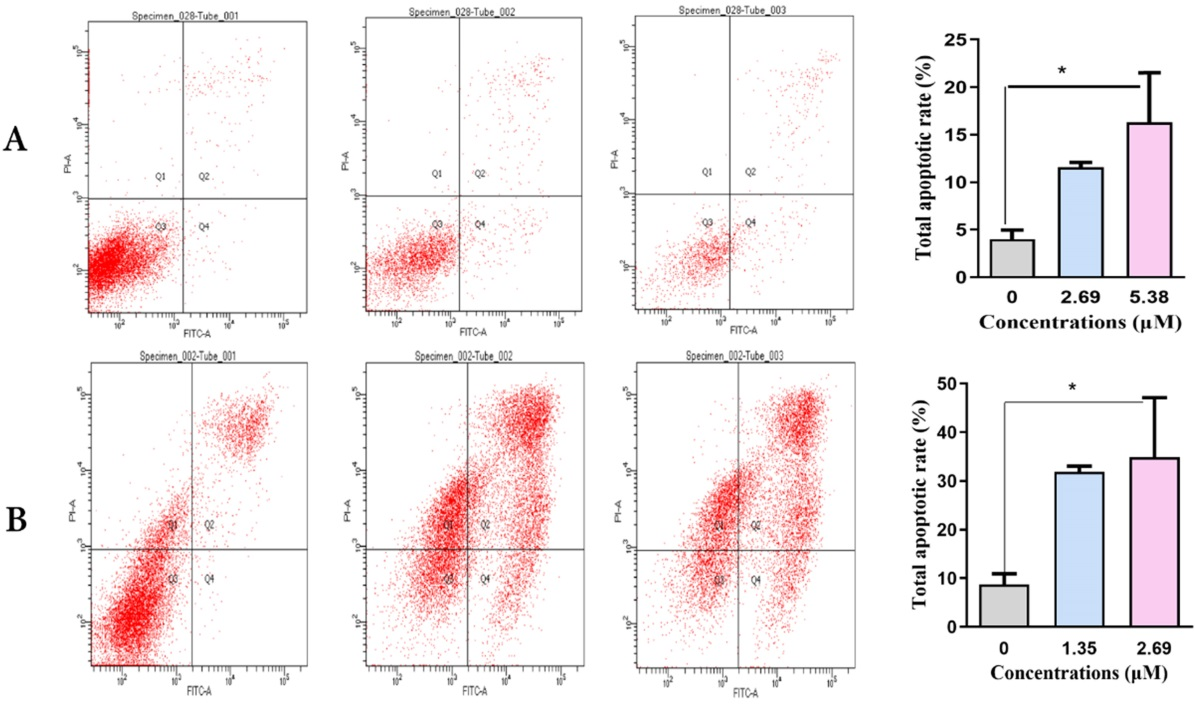
The total apoptotic rates of NSCLC cells increased in line with increasing SNX-2112 concentrations. Total apoptotic rates of A549 cells (A) and H520 cells (B) were examined using an Annexin V-FITC/PI staining assay after incubation with SNX-2112 for 48 h (^*^P<0.05).

Next, we detected the capacity of various concentrations of SNX-2112 to identify whether SNX-2112 could induce the apoptosis of A549 and H520 NSCLC cells over 48 h using flow cytometry. The total apoptotic rates of A549 (**Figure 3A**) and H520 cells (**Figure 3B**) increased significantly (both P<0.05) in line with increasing SNX-2112 concentrations (**Table 2**). Together, these findings indicate that SNX-2112 effectively induced apoptosis *in vitro*.

### SNX-2112 inhibited NSCLC cell invasion and migration

**Figure 4:**
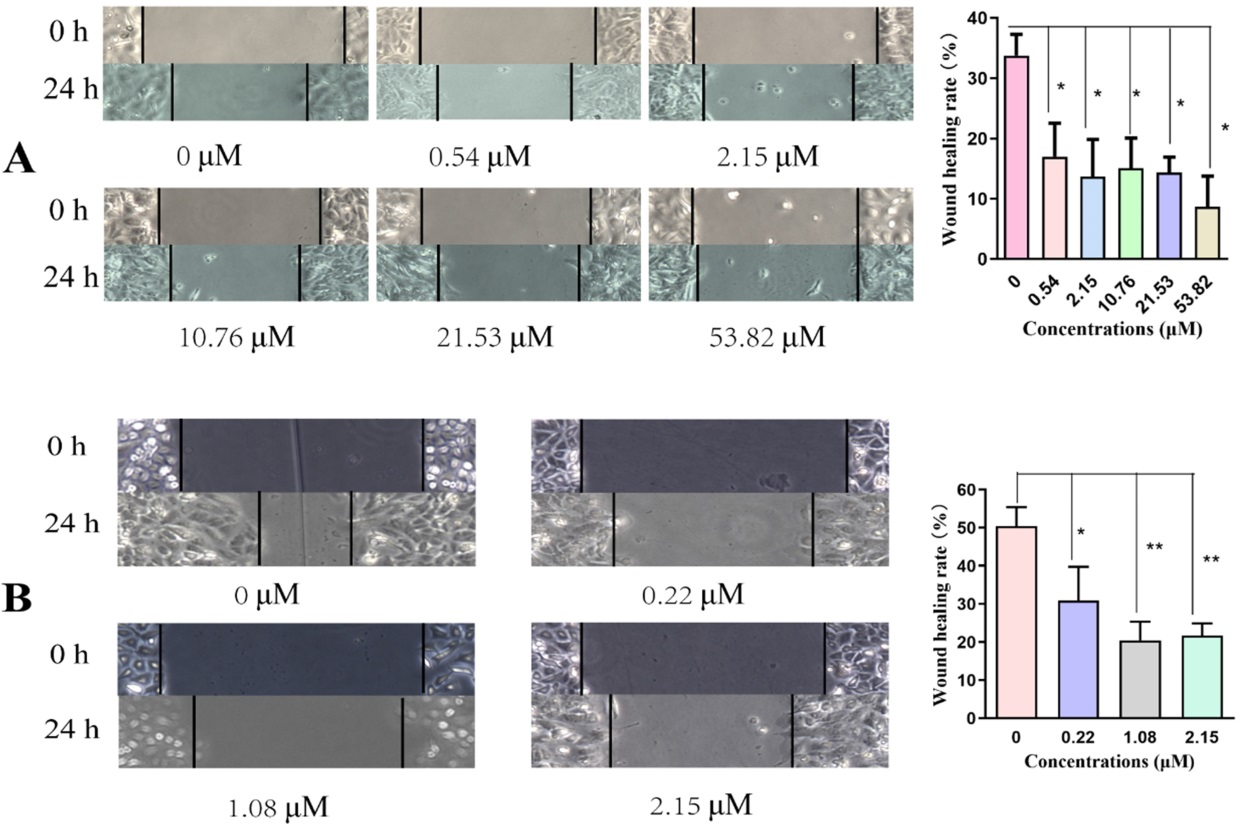
Cell migration was reduced by treatment with various concentrations of SNX-2112. The effects of SNX-2112 on the migration abilities of A549 cells (A) and H520 cells (B) were determined by a wound-healing assay (^*^P<0.05; ^**^P<0.01).

**Figure 5:**
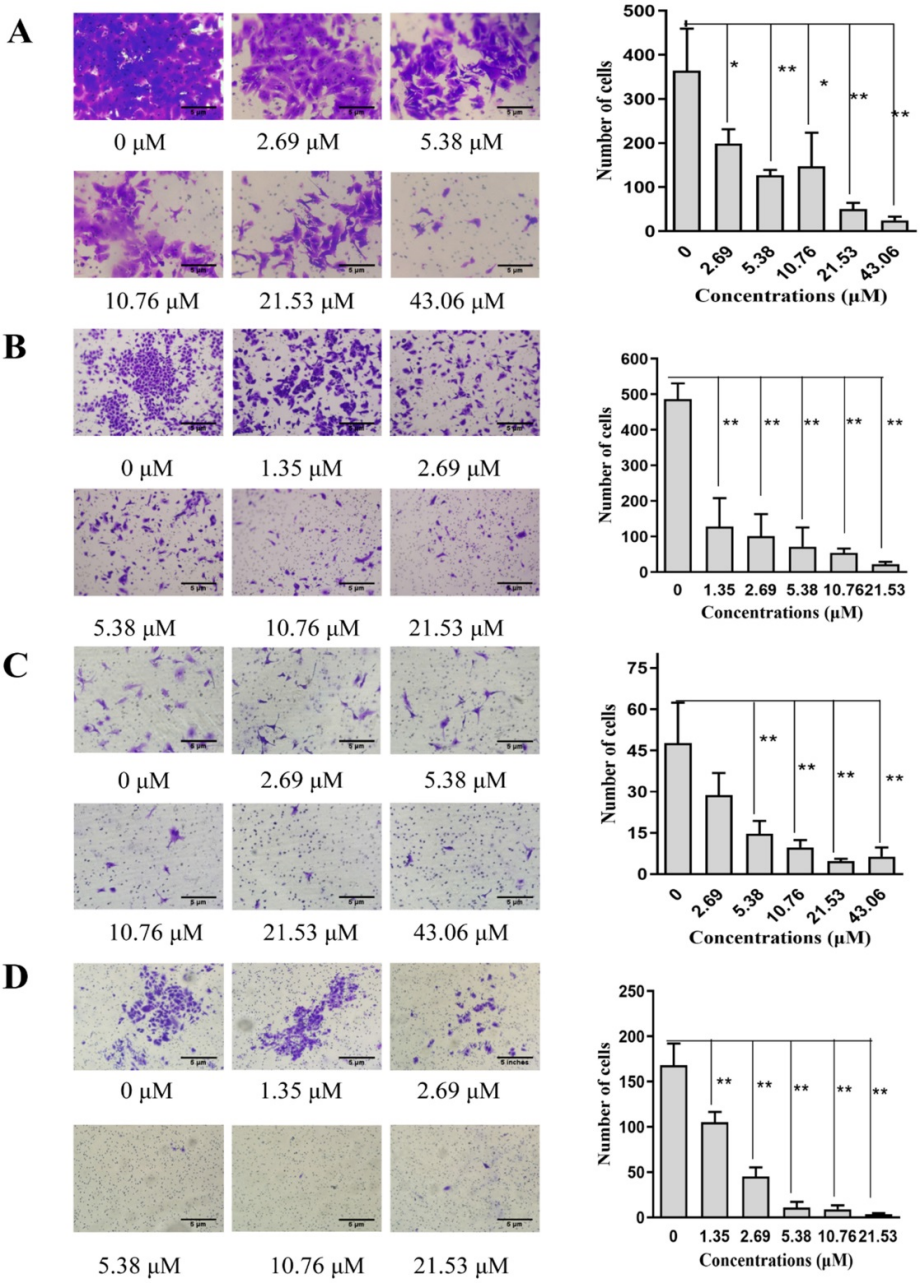
SNX-2112 inhibited cell invasion and migration. The migration abilities of A549 (A) and H520 cells (B) and the invasion abilities of A549 (C) and H520 cells (D) were all detected by a Transwell assay (^*^P<0.05; ^**^P<0.01). Magnification times of all images: 100x.

We used a wound-healing assay to explore cell migration. A Transwell assay was then used to detect the level of cell motility and invasion. Cell migration was significantly reduced in a concentration-dependent manner after incubating with SNX-2112 for 24 h, indicating that SNX-2112 inhibited the migration of A549 (P<0.05) and H520 cells (P<0.05 and P<0.01) (**Figure 4A and B**). As expected, the NSCLC cell migration and invasion capacities were also reduced with elevating concentrations of SNX-2112 (**Figure 5A-D**). These results revealed that SNX-2112 suppressed cellular invasion and migration *in vitro* (P<0.05 and P<0.01).

### SNX-2112 inhibited the EMT procedure in NSCLC

**Figure 6:**
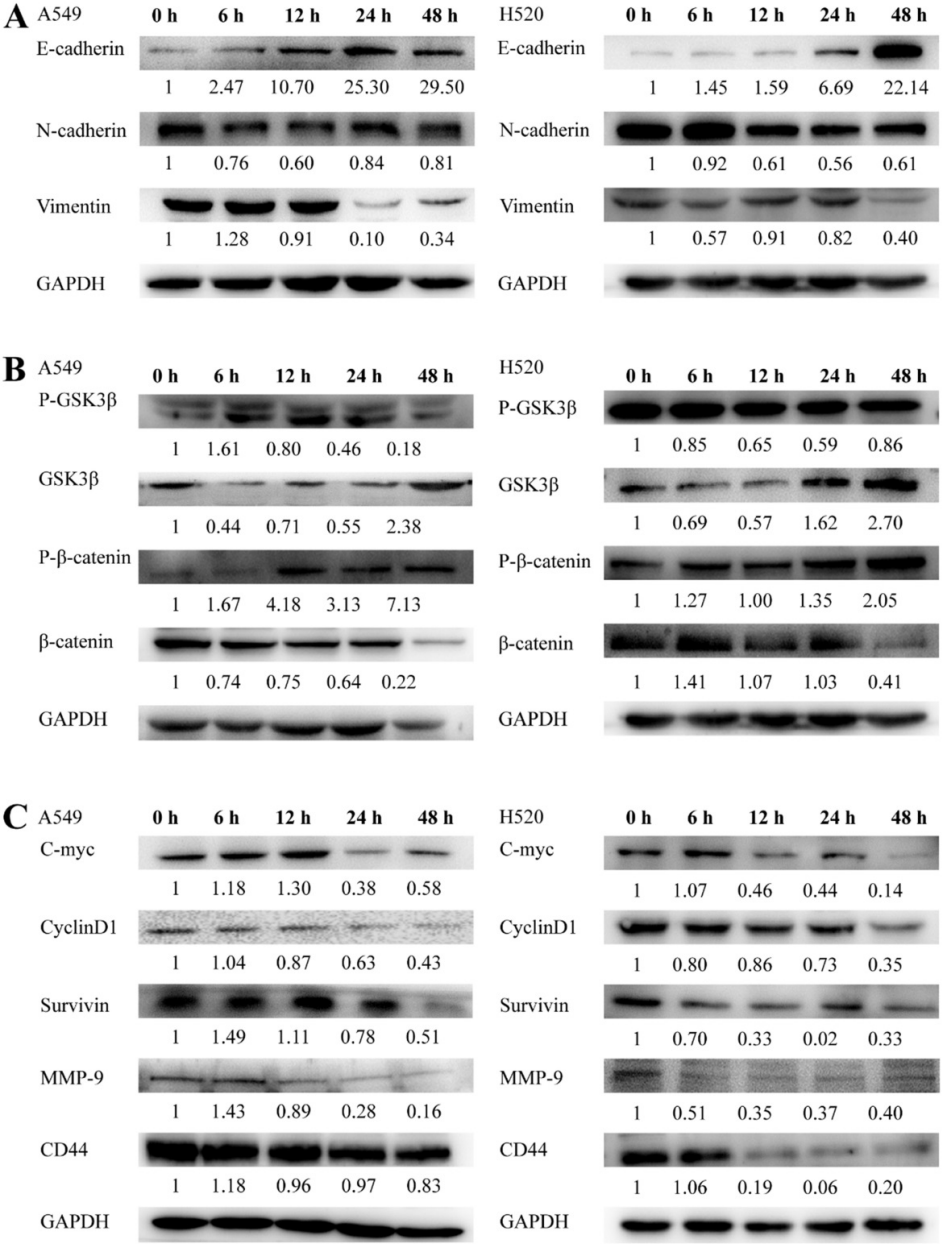
SNX-2112 inhibited EMT in NSCLC cells. A549 and H520 cells were treated with 21.53 µM SNX-2112 for 0, 6, 12, 24, and 48 h. (A) E-cadherin expression increased whereas the expression of N-cadherin and vimentin decreased in both cell lines. (B) SNX-2112 suppressed the Wnt/β-catenin pathway. The GSK3β and p-β-catenin expression increased while the β-catenin and p-GSK3β decreased in both cell lines. (C) SNX-2112 inhibited protein expression levels of downstream genes in the Wnt/β-catenin pathway. MMP-9, C-Myc, cyclinD1, CD44, and survivin expression levels were decreased in both cell lines.

To assess the mechanism by which SNX-2112 may affect NSCLC cells, EMT-related proteins were detected using a Western blot assay. As the bands revealed, the protein expression of E-cadherin was enhanced whereas that of N-cadherin and vimentin was reduced in both cell lines (**Figure 6A**), indicating that SNX-2112 inhibited EMT in NSCLC cells.

### SNX-2112 inhibited the Wnt/β-catenin signaling pathway

Given that the mechanism by which SNX-2112 affected NSCLC cells remained ambiguous, we next inspected the level of expression of the proteins in the Wnt/β-catenin pathway in NSCLC cells with the administration of 21.53 µM SNX-2112 for 0, 6, 12, 24, and 48 h. GSK3β and p-β-catenin were significantly increased in both cell lines, whereas β-catenin and p-GSK3β were decreased (**Figure 6B**). Overall, the results indicate that SNX-2112 inhibited the Wnt/β-catenin pathway to some extent.

### SNX-2112 inhibited downstream gene expression in the Wnt/β-catenin pathway

Next, we clarified the mechanism underlying the effect of SNX-2112 by observing and analyzing the experimental outcomes of the downstream genes within the Wnt/β-catenin pathway. In brief, we observed that the level of MMP-9, C-Myc, cyclinD1, CD44, and survivin were decreased by treatment with 21.53 µM SNX-2112 (**Figure 6C**). Thus, these findings implied that SNX-2112 inhibited the expression of the downstream genes via the Wnt/β-catenin pathway.

### Rescue experiments

The Wnt/β-catenin pathway agonist, SKL2001, can disrupt the Axin/β-catenin interaction, which is a critical step in the casein kinase 1 (CK1) and GSK-3β-mediated phosphorylation of β-catenin at Ser33/37/Thr41 and Ser45 23, 24. SKL2001 upregulated β-catenin-responsive transcription by increasing the intracellular levels of β-catenin protein and inhibiting its phosphorylation at Ser33/37/Thr41 and Ser45, which would otherwise mark it for proteasomal degradation, without affecting CK1 and GSK-3β enzyme activities 23, 24.

**Figure 7:**
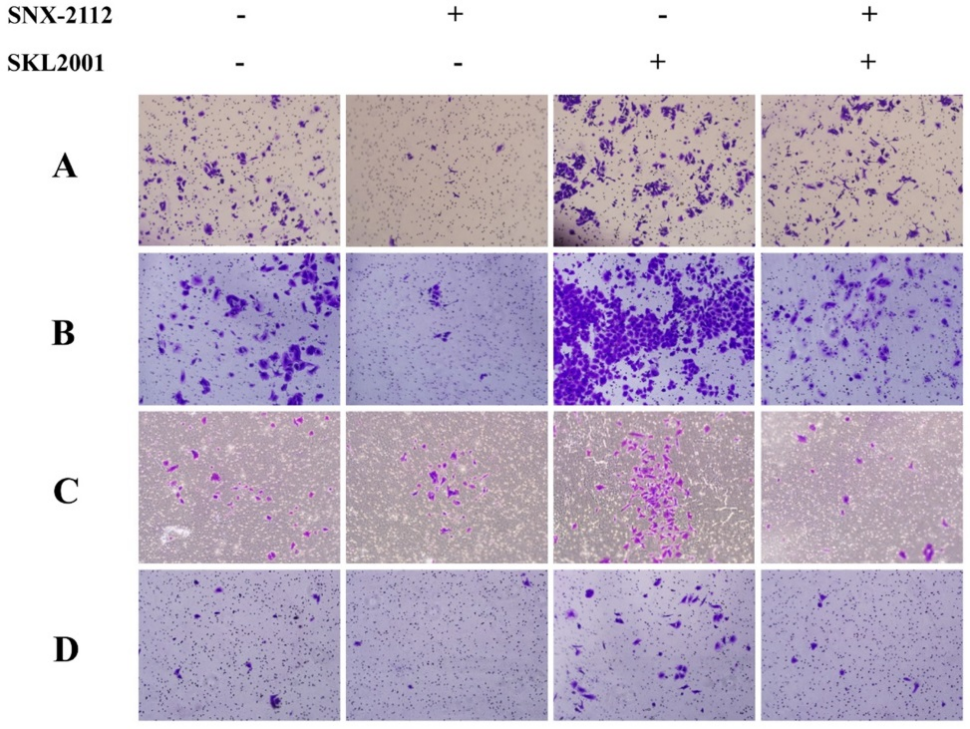
The prohibitive effect of invasion and migration was offset by SKL2001. The migration (A) and invasion (C) abilities of A549 cells treated with SNX-2112 (5.38 µM) and SKL2001 (20 µM) and the migration (B) and invasion (D) capacity of H520 cells treated with SNX-2112 (1.35 µM) and SKL2001 (20 µM) were assessed by a Transwell assay. Magnification times of all images: 100x.

**Figure 8:**
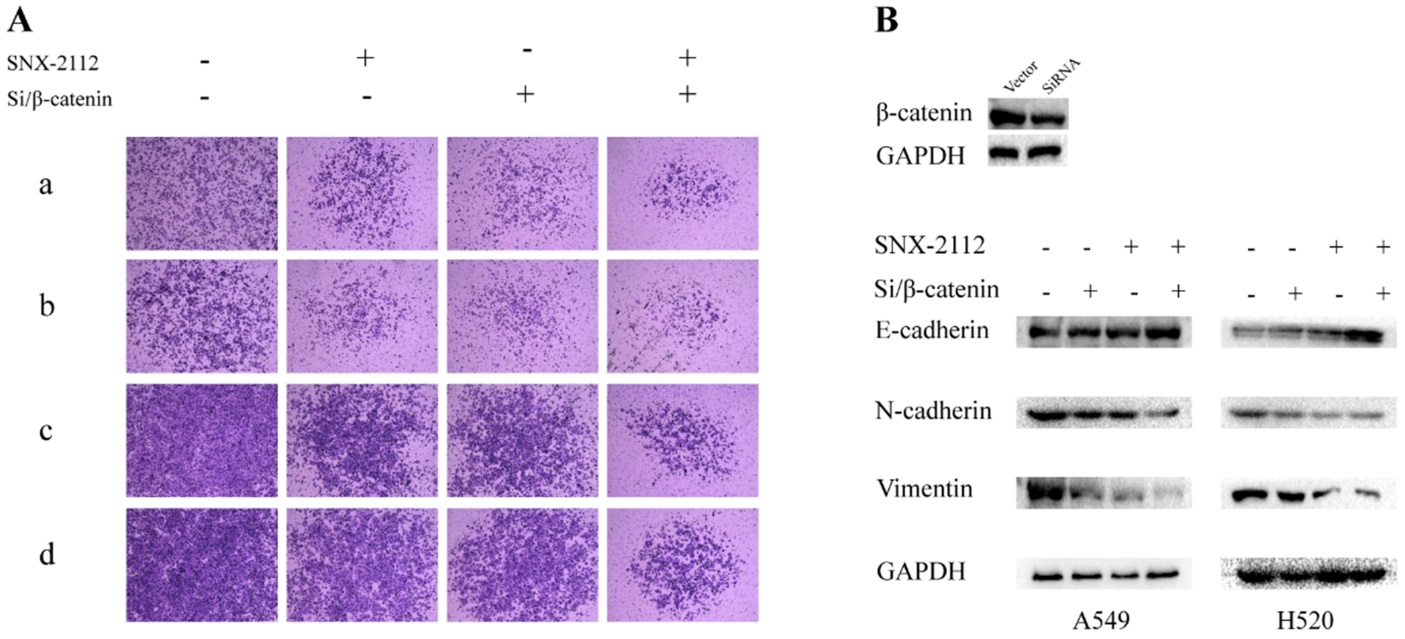
SNX-2112 suppressed EMT via Wnt/β-catenin pathway. (A) The migration (a) and invasion (b) abilities of A549 cells treated with SNX-2112 (5.38 µM) and SiRNA of β-catenin and the migration (c) and invasion (d) capacity of H520 cells treated with SNX-2112 (1.35 µM) and SiRNA of β-catenin were assessed by a Transwell assay. Magnification times of all images: 50 x. (B) The expression of N-cadherin and vimentin were decreased, while the expression of E-cadherin was increased in the SNX-2112 plus β-catenin knockdown group, compared to the β-catenin knockdown only group.

In the rescue experiment, SNX-2112 suppressed the invasion and migration of both A549 and H520 cells, and the prohibitive effect was partially offset by SKL2001 (**Figure 7**). Knockdown of β-catenin inhibited the invasion and migration of A549 and H1299 cells, and the suppressive effect became more pronounced after administration with SNX-2112 (**Figure 8A**). Meanwhile, we performed western blot experiments to further illustrate the mechanism of SNX-2112. And the results showed that the expression of N-cadherin and vimentin was decreased, while the expression of E-cadherin was increased in the β-catenin knockdown group and the SNX-2112 treated group, which indicating that the process of EMT was suppressed. When SNX-2112 was added to β-catenin knockdown cells, the inhibition of EMT was even more evident (**Figure 8B**). These results further verified that SNX-2112 suppressed the ability of NSCLC cells to invade and migrate by inhibiting the Wnt/β-catenin pathway.

### SNX-2112 suppressed tumor growth

**Figure 9:**
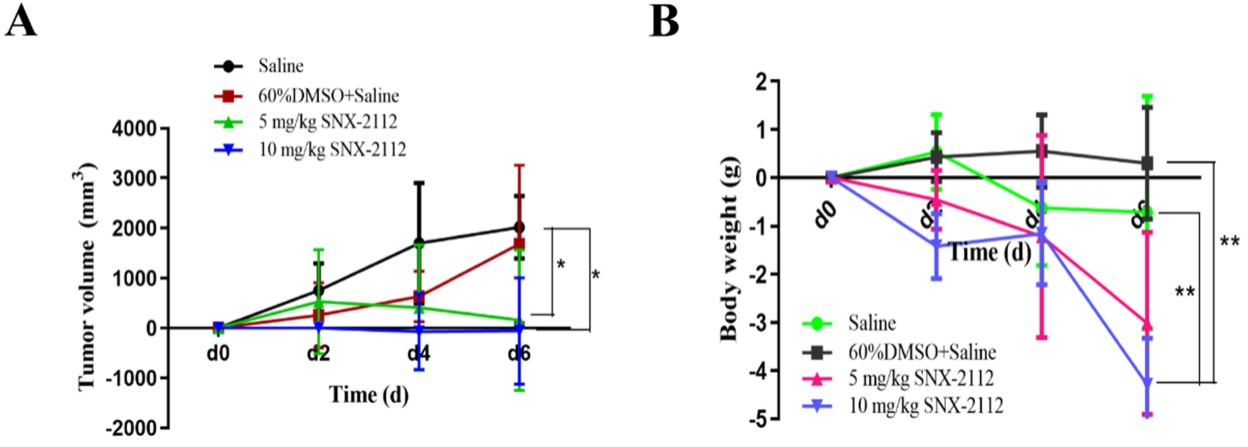
SNX-2112 suppressed tumor growth. Male BALB/c nude mice were injected subcutaneously with 5.5×10^6^ A549 cells/100 µL and treated with saline, saline plus 60% DMSO, low-dose (5 mg/kg) SNX-2112, or high-dose (10 mg/kg) SNX-2112. (A) Tumor volume curves in xenograft model mice (*P<0.05). (B) Mouse body weight recording (^**^P<0.01).

The tumor volume was calculated, and tumor-volume curves were plotted. In comparison with the saline group, the tumor volume was substantially smaller in the 5 mg/kg and 10 mg/kg SNX-2112 groups (P<0.05) (**Figure 9A**). The tumors were also smaller in the 10 mg/kg SNX-2112 group compared with the 5 mg/kg SNX-2112 group, yet the P value was greater than 0.05, which indicated that the difference was not significant (**Figure 9A**). Meanwhile, mouse body weight dropped by a large margin in the 10 mg/kg SNX-2112 group compared with the saline group and the 60% DMSO group (**Figure 9B**) (P<0.01).

### SNX-2112 promoted tumor tissue necrosis and tumor cell apoptosis

**Figure 10:**
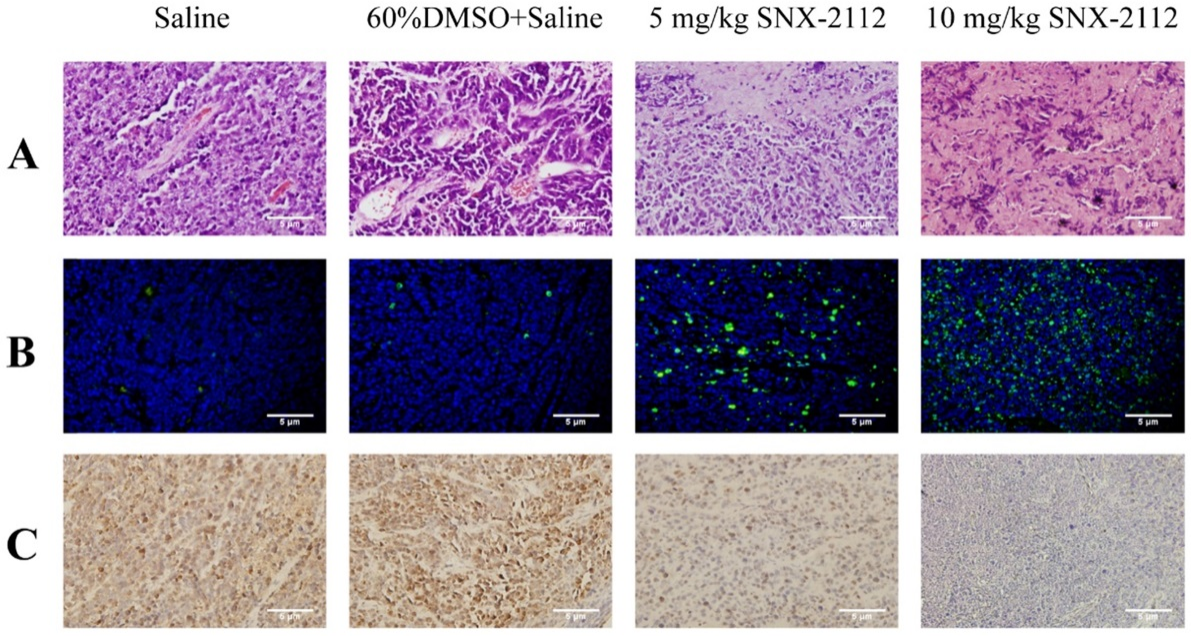
Tumor tissue specimens from xenografted mice were subjected to HE, TUNEL, and IHC staining. (A) HE staining showed that SNX-2112 promoted tumor tissue necrosis. (B) TUNEL staining showed that SNX-2112 promoted tumor cell apoptosis. (C) IHC staining showed that Ki-67 expression was reduced in the SNX-2112 high-dose group (10 mg/kg) compared with the low-dose (5 mg/kg), saline, and saline plus 60% DMSO groups, indicating that SNX-2112 inhibited tumor cell proliferation *in vivo*. Magnification times of all images: 100x.

The tumors were dissected from the mice after sacrifice (**Figure 9**) and subjected to HE staining to evaluate the degree of necrosis in each group. Tumor tissue necrosis was increased in the mice treated with 5 mg/kg or 10 mg/kg SNX-2112 compared with the normal saline and normal saline plus 60% DMSO groups (**Figure 10A**). Furthermore, the rate of necrosis was higher in the SNX-2112 high-dose in comparison with the low-dose group. In addition, we set up the saline group containing 60% DMSO as a control on account of the insoluble property of SNX-2112. The results suggested that there was no difference in tissue necrosis between the saline group containing 60% DMSO and the saline group.

A TUNEL assay was performed to detect apoptotic tumor tissue. In comparison with the low-dose group, the level of apoptosis was higher in the high-dose group (**Figure 10B**). The IHC results were not analyzed statistically but suggested that SNX-2112 inhibited tumor cell proliferation in accordance with the *in vitro* results (**Figure 10C**).

## Discussion

SNX-2112 was previously shown to induce apoptosis in multidrug-resistant K562/ADR cells through the suppression of Akt/nuclear factor-κB and disruption of mitochondria-dependent pathways 25. SNX-2112 also inhibited the proliferation of esophageal cancer cells by regulating the expression of excision repair cross-complementing 1, epidermal growth factor receptor, and p53 26. SNX-2112 exhibited potent anticancer activity against B16 melanoma cells both *in vitro* and *in vivo* by inhibiting cell proliferation and inducing cell cycle arrest and apoptosis via a mechanism involving the degradation of Hsp90 client proteins 22. However, these previous reports did not discuss the effects of SNX-2112 in NSCLC, nor did they clarify the underlying mechanism of cell invasion and migration. Based on the multiple experiments that have been conducted, we verified that SNX-2112 suppressed the proliferation, invasion, and migration, as well as boosted the apoptosis of A549 and H520 NSCLC cells *in vitro*. We used SKL2001 to further validate that SNX-2112 might suppress cell proliferation through inhibiting the Wnt/β-catenin pathway. The effect of SKL2001 in cell apoptosis will be verified in future studies. Moreover, SNX-2112 also suppressed the proliferation and induced the apoptosis of NSCLC cells in xenografted mice *in vivo*, consistent with the *in vitro* results. Similarly, an analog of SNX-2112 was also found to inhibit tumor growth in a mutant EGFR NSCLC model 27. To our knowledge, SNX-2112 has not been studied in NSCLC. In addition, the protein analysis showed that GSK3β and p-β-catenin protein expression levels were increased in NSCLC cells after treatment with SNX-2112, while β-catenin and p-GSK3β levels were decreased, suggesting that SNX-2112 might inhibit the invasion and migration of NSCLC cells by inhibiting the Wnt/β-catenin pathway.

EMT is a cellular process by which epithelial cells transform into mesenchymal cells. EMT is induced by a variety of growth factors, including transforming growth factor-β, hepatocyte growth factor, insulin-like growth factor, and epidermal growth factor 28, 29. E-cadherin expression is repressed after EMT activation, resulting in the loss of typical epithelial polygonal and cobblestone morphology. The cells then acquire a fusiform mesenchymal morphology and overexpress mesenchymal markers, including N-cadherin, vimentin, and fibronectin 30, 31. Activation of EMT can also lead to the disruption of cell-to-cell junctions, degradation of the underlying basement membrane, and reorganization of the extracellular matrix. Furthermore, activation of the EMT process in carcinoma cells can enhance cell viability, as well as increase their invasive capacity and resistance to apoptosis 31. EMT is also a well-studied procedure essential to tumorigeneses and metastases 32. Moreover, the aberrant activation of EMT is a critical mechanism for endowing epithelial cancer cells with the migratory and invasive capabilities associated with metastatic competence 33. Since SNX-2112 inhibited cell invasion and migration, we suspected that these effects were related to EMT. In the current study, E-cadherin expression was elevated in NSCLC cells following disposal with SNX-2112, whereas N-cadherin and vimentin were decreased. This suggested that SNX-2112 could reverse the occurrence of EMT, thereby inhibiting cell invasion and migration.

EMT was recently considered to be an early event in tumor invasion and metastasis 34. Although multiple signaling pathways are related to EMT, the specific molecular mechanism remains ambiguous. Wnt/β-catenin is a promising signaling pathway related to tumor invasion and metastasis 35 and promotes tumor progression by regulating EMT and cancer stem cells 36. E-cadherin is a negative regulator of the Wnt pathway and loss of E-cadherin, largely influenced by Wnt/β-catenin signaling, which is a major contributor to the EMT process 37. The dysregulation of Wnt plays a critical role in inducing the transcription factor, Snail, in EMT 38. β-Catenin also binds to the SLUG, ZEB 39, and TWIST promoters 40, as well as facilitate their expression. β-Catenin expression is regulated by various factors and in turn regulates the induction of EMT 40. P53 activates the transcription of miR-200 family members through a mutually regulatory feedback loop, and miR-200 expression is also related to ZEB1 and Snail, respectively 39. Recent studies have shown that EMT was inhibited by cyclin G2 via Wnt/β-catenin signal transduction 41. Both ovarian and breast cancer studies have presented evidence of the mechanism of Wnt/β-catenin pathway activation and Wnt signaling-mediated promotion of EMT 42, 43. While our study did not show the specific process, we did verify that SNX-2112 inhibited downstream gene expression in the Wnt/β-catenin pathway. Specifically, we tested the level of MMP-9, C-Myc, cyclinD1, CD44, and survivin and found that the level of protein expression was decreased following SNX-2112 treatment. This finding further revealed the relationship between SNX-2112 and the Wnt/β-catenin pathway. Given that the Wnt/β-catenin pathway is involved in the EMT process, we speculated that SNX-2112 might reverse the Wnt/β-catenin pathway and EMT, eventually inhibiting the invasion and migration of NSCLC cells. To verify this hypothesis, we administrated SNX-2112 to β-catenin knockdown for Transwell assays, and the results indicated that SNX-2112 could diminish the migration and invasion abilities of β-catenin knockdown cells. And the Western Blot assays showed that the expression of E-cadherin was enhanced, while the expression of N-cadherin and vimentin was attenuated compared to the SNX-2112 group and the β-catenin knockdown group. These results initially validated our assumption. For safety assessment of SNX-2112, normal human bronchial epithelial (NHBE) cells were applied for cytotoxicity tests and tissue damage assays of GO-CHI-HA/SNX-2112 44. These data have confirmed that SNX-2112 is safe and suitable for clinical application.

In conclusion, although this research revealed that SNX-2112 suppressed both EMT and the Wnt/β-catenin pathway, the specific mechanism responsible for this process remains obscure, and further research is therefore required to resolve this issue. It has been established that this study provides the first evidence for the mechanism of SNX-2112 in NSCLC. Hence, the results indicated that SNX-2112 could induce apoptosis and inhibit proliferation, invasion, and migration of NSCLC cells by downregulating EMT via the Wnt/β-catenin pathway. Furthermore, our research offers a potentially useful molecule as a clinical therapeutic for NSCLC.

## Figures and Tables

**Table 1:** IC50 values of SNX-2112 in NSCLC cells

	IC50 value (μM)		
	24h	48h	72h
A549	148.01±18.99	13.67±10.76	0.45±0.04
H520	152.30±8.57	2.07±1.72	1.72±1.50

**Table 2:** Total apoptotic rates of NSCLC cells following the administration of various concentrations of SNX-2112 (%)

	NC	LCG	P1	HCG	P2
A549	4.03±0.02	11.57±0.01	0.134	16.30±0.09	0.030*
H520	8.73±0.04	32.00±0.02	0.064	34.83±0.21	0.043*

(* P<0.05) NC, negative control; LCG, low concentration group; HCG, high concentration group; P1, P value1; P2, P value2.

## Abbreviations

NSCLC: non-small cell lung cancer
EMT: epithelial-mesenchymal transition
Hsp90: heat shock protein 90
p-β-catenin: phosphorylated-β-catenin
GSK3β: glycogen synthase kisnase3β
p-GSK3β: phosphorylated-glycogen synthase kinase3β
MMP-9: matrix metalloproteinase-9
GAPDH: glyceraldehyde 3-phosphate dehydrogenase
CK1: casein kinase 1
FBS: fetal bovine serum
PBS: phosphate-buffered saline
